# An effective brain stroke diagnosis strategy based on feature extraction and hybrid classifier

**DOI:** 10.1038/s41598-025-14444-8

**Published:** 2025-08-14

**Authors:** Maha Samir Elsayed, Gehad Ahmed Saleh, Ahmed I. Saleh, Abeer Twakol Khalil

**Affiliations:** 1 Department of Biomedical Engineering, Faculty of Engineering, Mansoura, Egypt; 2 Department of Diagnostic and Interventional Radiology, Faculty of Medicine, Mansoura, Egypt; 3 Department of Computers and Control System Engineering, Faculty of Engineering, Mansoura, Egypt; 4 Department of Electronics and Communications Engineering, Faculty of Engineering, Mansoura, Egypt

**Keywords:** Brain stroke diagnosis, Effective brain stroke diagnosis strategy (EBDS), VGG16, Vision transformer (ViT), Feature extraction, Feature fusion, Hybrid classifier, Medical imaging, Biotechnology, Computational biology and bioinformatics, Diseases, Engineering

## Abstract

Stroke is a leading cause of death and long-term disability worldwide, and early detection remains a significant clinical challenge. This study proposes an Effective Brain Stroke Diagnosis Strategy (EBDS). The hybrid deep learning framework integrates Vision Transformer (ViT) and VGG16 to enable accurate and interpretable stroke detection from CT images. The model was trained and evaluated using a publicly available dataset from Kaggle, achieving impressive results: a test accuracy of 99.6%, a precision of 1.00 for normal cases and 0.98 for stroke cases, a recall of 0.99 for normal cases and 1.00 for stroke cases, and an overall F1-score of 0.99. These results demonstrate the robustness and reliability of the EBDS model, which outperforms several recent state-of-the-art methods. To enhance clinical trust, the model incorporates explainability techniques, such as Grad-CAM and LIME, which provide visual insights into its decision-making process. The EBDS framework is designed for real-time application in emergency settings, offering both high diagnostic performance and interpretability. This work addresses a critical research gap in early brain stroke diagnosis and contributes a scalable, explainable, and clinically relevant solution for medical imaging diagnostics.

## Introduction

Stroke is among the most alarming health issues worldwide, being a leading cause of death and permanent disability. According to the Centers for Disease Control and Prevention (CDC), strokes account for around 17.5% of deaths from cardiovascular diseases in the United States, while every 40 s, a person experiences a stroke^1^. The incidence of stroke has been increasing, mainly ischemic and hemorrhagic, despite all medical research and therapeutic advancements. The burden of stroke comprises not only clinical but also economic and social aspects, having an impact on millions of individuals and health-care systems worldwide. In contrast, early and accurate diagnosis can significantly improve the prognosis for patients, and timely intervention may prevent fatal complications.

Although early diagnosis of stroke is critical for maintaining the prognosis, conventional diagnostic methods still face direct limitations in reliability. Some diagnostic processes involving CT or MRI tests rely heavily on manual interpretation performed by radiologists or neurologists. While these imaging techniques assist in identifying the type and location of stroke incidents, the manual review often becomes a lengthy, exhausting process, subject to potential human error, which can delay crucial decision-making in emergencies. Ischemic strokes may not present clear signs on CT during the first hours of onset, thereby delaying treatment with adverse effects on the patient^2^. These limitations have driven the exploration of AI-based solutions to support clinical decision-making. The health scenarios attribute an urgency to the solutions for diagnostics that are fast, accurate, and run automatically, thereby supporting clinicians in their timely and reliable decision-making. AI in medical imaging offers revolutionary opportunities for brain stroke diagnosis. Among AIs, deep learning stands out with top performance for analyzing complicated medical images with high accuracy and speed^3^. Among the various deep learning models explored for medical imaging, an RNN architecture will provide the greatest performance when the structure of the data is sequential, as in the case of EEG and ECG signals, where the temporal dependencies matter most^4^. Conversely, an RNN performs poorly on static images, with problems occurring in multiple algorithmic steps, e.g., gradient vanishing owing to the backpropagation through time (long gradient chains) and the inability to appropriately change spatial features. Another class of models, autoencoders, has been applied in unsupervised tasks such as anomaly detection, image denoising, and processing. They learn compressed representations of the input data, which may be useful in the detection of minute abnormalities. They learn compressed representations of the input data, which may be useful in the detection of minute abnormalities. However, because of the lack of interpretability behind the autoencoder, its outputs are mostly uninterpretable to medical professionals, which in turn limits the clinical applicability of such methods. The most useful applications of generative adversarial networks have been to create synthetic medical images and augment limited data sets. This is especially valuable in domains where annotated data is scarcely available. However, while GANs are powerful, their training may become unstable, leading to inconsistent results if a neat training regimen is not met^5^. While each of these models has advantages, its disadvantages strengthen the need for a combination of complementary architectures toward more robust and clinically useful diagnostic systems. Among the most widely used deep learning models are CNNs, more efficient in extracting spatial features from CT and MRI scans. Because of their amazing capability of learning hierarchical representations of image data, CNNs have found their way into brain stroke diagnosis, tumor detection, and organ segmentation^6^. However, the major challenge remains in capturing long-range interactions and global contextual information in brain imaging, which are essential for detecting subtle abnormalities. In response to this challenge, ViTs have quickly ascended as an excellent alternative. Unlike CNNs, ViTs use self-attention mechanisms to allow the model to utilize context from the most relevant regions of the image for recognizing complex and fine-grained patterns. Thus, ViTs are apt for the detection of early stroke, in which slight changes in brain tissue may be hard to identify through conventional means^7^. Recent works outline the superiority of ViTs to CNNs for a number of medical imaging tasks, particularly when trained on sufficiently large and diverse sets of data^8^. Besides CNNs and ViTs, several other architectures of deep learning, though largely unproven so far in medical applications, promise to hold potential. As brain stroke diagnosis involves a medical imaging domain, there are several types of diagnoses performed, one per model type. The ability of these models to simultaneously capture local and global image features is limited, despite the fact that they perform well when used alone. To get around this restriction, complementary models must be integrated^9^. Combining ViT and VGG16 into an ensemble deep learning model is a promising approach that combines ViT’s global contextual awareness with VGG16’s potent local feature extraction capabilities. This integration is expected to result in higher diagnostic accuracy and faster inference times for brain stroke diagnosis from medical imaging when compared to using either model independently. To address these limitations, this study proposes a hybrid deep learning framework, EBDS, that combines the strengths of ViT and VGG16 to improve early brain stroke diagnosis accuracy and efficiency.

In light of these challenges and the limitations of existing diagnostic models, this study proposes a hybrid deep learning framework that integrates ViT and VGG16 to enhance both the accuracy and interpretability of brain stroke diagnosis from CT images. The research is driven by the hypothesis that significantly improves diagnostic performance over using either model in isolation. To validate this hypothesis, the study explores whether the proposed hybrid model can outperform individual architectures in stroke classification, evaluates its performance across multiple metrics, including accuracy, precision, recall, and F1-score, and examines its clinical relevance as an interpretable AI tool. Furthermore, the model’s effectiveness is benchmarked against recent state-of-the-art approaches to assess its comparative advantage.

**The main contribution of this study is it’s as follows**

(i) **Novel classification method:** The paper proposes a new approach for classifying brain stroke disease that demonstrates robust performance across datasets, emphasizing the importance of interpretability and explainability for clinical applications.
(ii) **Advanced integration of neural networks:** This study integrates features from ViT and VGG16 to create a comprehensive and informative feature representation. This fusion leverages the strengths of both architectures, resulting in superior performance compared to individual models.
(iii) **Enhanced model performance:** By combining ViT and VGG16, the paper achieved improved discriminative ability, capturing a broader range of visual features and surpassing the performance of the individual models.

The remainder of this paper is structured as follows. Section "Problem definition and suggested solution" introduces the Problem Definition and Suggested Solution. Section "Related works" presents the Related Works in AI-based brain stroke diagnosis. Section "Methodology" describes the Methodology, including dataset preparation, preprocessing, feature extraction, and fusion. Section "Results" reports the Results of the proposed model. Section "Discussion" provides a detailed Discussion on interpretability and clinical relevance. Section "Model performance evaluation" presents the Model Performance Evaluation, including comparisons and ablation studies. Section "Limitations" outlines the Limitations of the study. Finally, Sect. "Conclusion and future work" concludes and Future Work.

## Problem definition and suggested solution

**Fig. 1:**
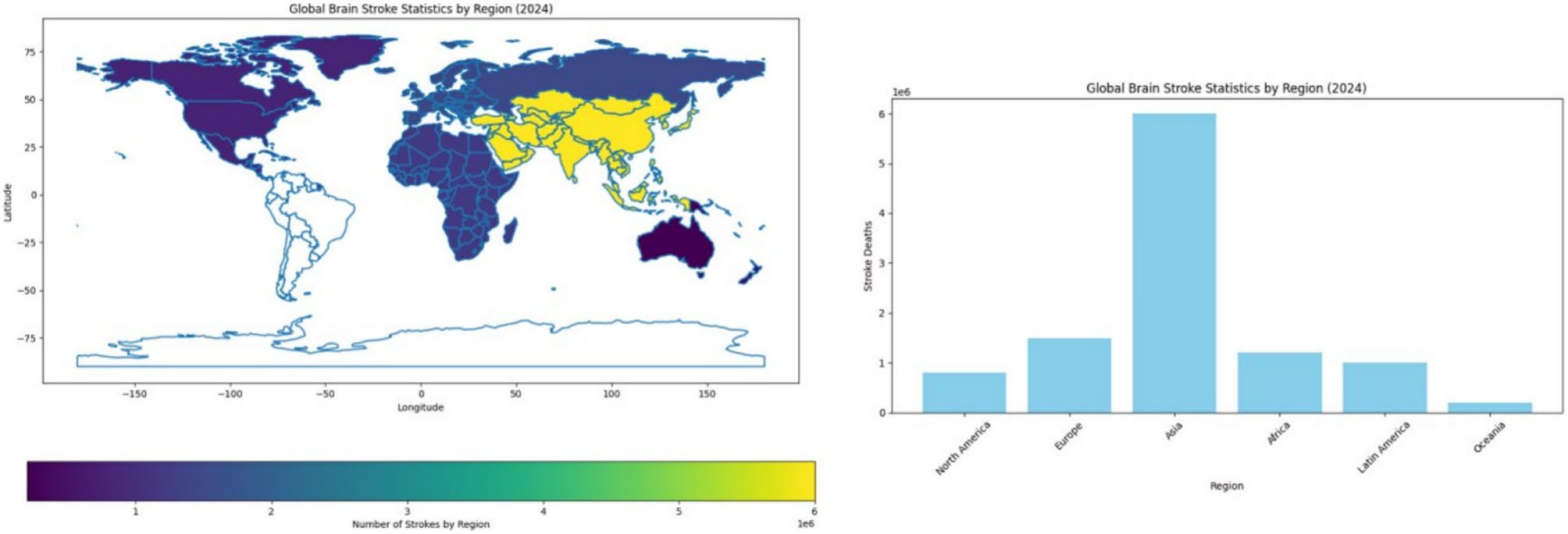
Global distribution of stroke incidences by region.

**Fig. 2:**
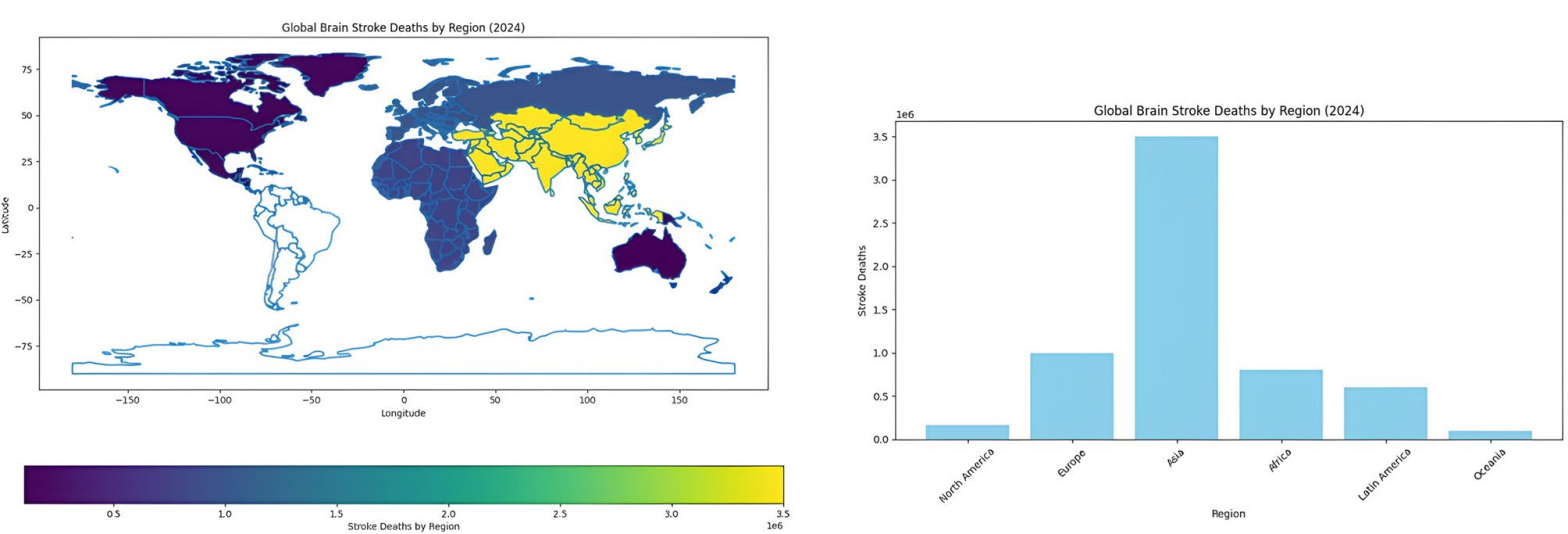
Global distribution of stroke-related deaths by region.

Finding strokes in human brains with accuracy and at an early enough point in time is a problem faced in medicine. Symptoms of stroke vary a lot with different individuals and may sometimes present subtly or mimic the symptoms of other conditions, causing hindrance in their early detection. Imaging techniques like CT and MRI are effective but might miss some very early signs of stroke or fail to provide enough information to arrive at a definite diagnosis. Opportunity for the treatment is time-dependent, where any delay in diagnosis would cause increased brain damage and a lesser chance for recovery. Likewise, the patterns of patients and stroke types might differ substantially from one case to another, making it even more difficult to formulate a common tool for the diagnosis of any stroke under all possible circumstances and situations. Conceptually, combining imaging, clinical record data, genetic factors, etc., to generate a comprehensive diagnostic model is difficult and expensive from the point of view of data processing^10,11^. As of 2024, stroke continues to be a chief public health issue worldwide. In the United States, every year, around 795,000 individuals suffer from a stroke, of which an estimated 610,000 are first or new strokes and approximately 185,000 are recurrent strokes. Globally, a major cause of death and disability, millions of new cases are reported every year. In the year 2021, stroke accounted for 162,890 deaths in the U.S., with an age-adjusted stroke death rate of 41.1 per 100,000. For the years 2019 and 2020, the stroke-related economic burden was almost $56.2 billion, which includes the cost of healthcare services, medications, and lost productivity. High blood pressure, high cholesterol, smoking, obesity, and diabetes are the top five stroke causes, with one out of every three adults in the U.S. having at least one of these conditions or risk factors. The risk of having a first stroke is almost twice as high for Black adults, and non-Hispanic Black and Pacific Islander adults hold the highest stroke death rates^12^. Figure 1 gives the world view of stroke occurrences by region in 2024. Stroke cases in Asia lead the world statistics with 6 million stroke cases every year. In stroke distribution terms, Europe and Africa follow. This also indicates a severe variation in different regions regarding stroke incidences and implies a need for an elevation of awareness and prevention work in the severely affected locations. The presentation of stroke death distribution in various regions in 2024 is laid out in Fig. 2. We see Asia to be the one with the most stroke deaths at 3.5 million deaths per year, followed by Europe and Africa. This distribution indicates the magnitude of health problems faced by certain regions and emphasizes the urgent task of improving the health sector and giving effective treatment to address such deaths. In this paper, the composite model of VGG16 and ViT that has been proposed in this paper can yield a more accurate and robust solution for the brain stroke diagnosis challenges. Although VGG16 is a convolutional neural network that understands the fine details of the spatial features of the image, the ViT framework serves to prune the long-distance dependencies and global context.

## Related works

Stroke is one of the most disabling and fatal causes throughout the world. Thus, it requires early and concise diagnosis to manage timely and efficient treatment. Most of the recent works have been aimed at developing computerized models that can identify strokes using advanced machine learning and deep learning techniques associated with medical imaging tools such as CT and MRI. Abulfaraj et al.^13^ incorporated SqueezeNet v1.1, MobileNet V3Small, and CatBoost as part of one deep learning model that is expected to achieve an accuracy of approximately 99.1%. The authors employed CT images to develop a very accurate model for brain stroke diagnosis. The proposed model faces challenges related to computational requirements and dependence on large datasets. Tursynova et al.^14^. Introduced a deep learning-based classifier for brain strokes from CT images, improving the overall model accuracy and recall by strategies such as data augmentation with flipping and early stopping. Even then, it reached only 79% model accuracy, reflecting that there could be more enhancement in the classes of performance. The practical feasibility of the study exists, but it fails in generalizing and implementing complexities. Ozaltin et al.^15^. Introduced a deep learning-based brain stroke diagnosis methodology from brain CT images using a novel type of CNN architecture, OzNet, along with several machine learning algorithms. This hybrid model achieved 98.42% accuracy with an AUC of 0.99, reflecting very strong performance, though it has challenges in its complexity and large volume data dependence. Chen et al.^16^. Introduced a deep learning-based model for detecting stroke using CT images, whose implementation can be developed to achieve high accuracy and robust performance using state-of-the-art methods. The authors present an architecture made of CNN-2, VGG-16, and ResNet-50 with 98.72% accuracy, which is optimized using hyperparameters and transfer learning to classify the brain CT images as normal, hemorrhage, infarction, and others. However, regarding computational complexity and heavy data requirements, it does pose some challenges that may make the implementation and maintenance of such systems cumbersome. Lo and colleagues^17^ aimed at classifying normal, ischemic stroke, and hemorrhagic stroke by developing a CNN for brain CT scans. The proposed model provides a highly automated, accurate classification that reduces manual interpretation and thus increases diagnosis speed. Besides, it is scalable, so it is able to perform even better when more data becomes available. However, model performance is very sensitive to the quality and size of data, and even computational resources, while model complexity can be prohibitive in interpretability, standing in the way of clinical adoption. Jayachitra and Prasanth^18^. Presented a stroke lesion segmentation approach by fuzzy segmentation algorithms, followed by classification using the weighted Gaussian Naive Bayes classifier. The results had a broad accuracy range in different datasets that varied from 30 to 99.32%. Highly versatile and accurate at maximum rate, this study enhances diagnosis due to proper identification and categorization of stroke lesions. However, the performance showed highly uneven rates, with an accuracy fluctuating from very high to very low in certain cases. Besides, a number of practical difficulties were presented: for instance, the complexity of the approach and dependence on data. Subuddhi et al.^19^ utilized MRI images and an algorithm to classify stroke subjects into three categories using the random forest classifier, which correctly classified 93.4%. The study, therefore, performed very well with quite effective classification, augmenting diagnostic precision by the robustness of random forest classifiers. However, challenges remain highly resource-demanding, computational demands, data dependency, and complex implementation and maintenance. Sabir et al.^20^ proposed a deep convolutional neural network (DCNN) model used to detect strokes in their very early stages. The model produces impressive accuracy results, achieving a remarkable 96.5% in detecting strokes in 6 h or less after onset, surpassing other models such as VGG16, ResNet50, and InceptionV3. It captures features from various layers so as to provide a full representation of the input image, thus improving patient management and outcomes. Nevertheless, the model does run into issues like complexity: the demand for vast amounts of computational resources, heavy dependence on the quality and diversity of the training data, and high interpretability rates, for which results are often hard to decipher owing to the complex nature of deep neural networks. Zafari-Ghadim et al.^21^ had done several works to examine the performance of different deep learning models for stroke segmentation. The study evaluates different architectures, such as the transformer-based, CNN-based, and hybrid architectures, as well as the self-adaptive nnUNet framework. The results indicate that the nnUNet framework achieved the best results with a much simpler design and stresses the importance of preprocessing and postprocessing techniques. However, on the downside are the DAE-Former and FCT, which are advanced models, computationally intensive, and on the quality of training data, which causes actual implementation challenges. Dubey et al.^10^ developed a systematic approach for predicting stroke patient survival with the assistance of machine learning. Optimized boosting algorithms, such as Gradient Boosting (GB), AdaBoost (ADB), and XGBoost, are utilized in the study, and ultimately, XGBoost was proved to be the best performer (with training accuracy standing at 96.97% and testing accuracy 92.13%). This model is characterized by high accuracy and the use of LIME and SHAP for explainability, thus allowing interpretation of its decisions. A downside, however, is that it is computationally intensive, extremely data-hungry with respect to quality and diversity of training data, and has a complex implementation and maintenance. Under the supervision of Subba Rao Polamuri and others, a brain stroke diagnosis framework was proposed using deep learning techniques on MRI images. The study implemented enhanced CNN architectures, namely DenseNet121, ResNet50, and VGG16, that were optimized exclusively for brain stroke diagnosis tasks. Those models were added to the supervised pipeline. Experimental results revealed that the proposed models performed much better than the baseline CNNs. Although promising, the study admits the need for excellent quality MRI as well as computational resources for a successful deployment^22^. A detailed survey of deep learning models for ischemic stroke lesion segmentation was presented by Khan et al. CNN, transformer, and hybrid models were reviewed, and the strengths of U-Net, nnU-Net, and attention-based networks were noted. The challenges of data imbalance in descriptions and the need for standardized evaluation metrics were emphasized^23^. Through the introduction of a brain stroke diagnosis problem within 6 h of onset, the model achieves an accuracy of 96.5%. Therefore, by Sabir and Ashraf, a novel deep convolutional neural network model was proposed for the early detection of brain stroke diagnosis using CT scan images. However, the model is rather one that needs very good quality CT data and is computationally intensive^20^. Abdi et al. developed a CNN model for brain stroke diagnosis using brain CT images. It had an accuracy of 97.2% during internal validation and 89.73% for the external dataset. It considered interpretability tools such as LIME, occlusion sensitivity, and saliency maps. The study pointed to further research to optimize these systems for improved generalizability^24^. Using facial and speech analysis of movement, Yu et al. developed a multimodal deep learning model for stroke symptom diagnosis to replicate clinical tools such as CPSS and FAST. It has a sensitivity of 93.12%, while the accuracy stands at 79.27%, and can fit within a smartphone for rapid self-assessment. The model requires accurate video input of movements in real-time, however^25^. Dhakan et al. implemented a classified learning approach in their overarching study to records indicating early stroke. An ensemble model performed better than a single classifier and boosting algorithms in the study. It is promising, yet there remain limitations considering dataset size and the requirement of clinical validation^26^. A 2025 study published by *Nature Biomedical Engineering* suggests a new deep learning system named DeepRETStroke that uses retinal fundus images rather than brain imaging to detect silent brain infarctions (SBIs) and predict stroke risk. The model was trained with more than 895,000 retinal images and obtained an AUC of 0.901 for an incident stroke prediction task. It Table 1 provides a summary of recent studies (2024–2025) on brain stroke diagnosis, highlighting the methods used, datasets employed, and reported performance metrics such as accuracy.

**Table 1:** Summary of brain stroke diagnosis studies using machine learning and deep learning (2024–2025).

Author	Year	Method	Dataset	Accuracy/performance	Dataset source/notes
Abdi et al.	2025	CNN with interpretability (LIME, saliency maps)	2501 CT images (internal), 9900 (external)	97.2% (internal), 89.73% (external)	Internal + external datasets
Yu et al.	2025	Multimodal DL (facial motion + speech)	Video data (CPSS/FAST emulation)	Sensitivity = 93.12%, Accuracy = 79.27%	Simulated/emulated dataset
Dhakan et al.	2025	Ensemble ML model on structured data	5110 patient records	Outperformed individual classifiers	Structured tabular data
DeepRETStroke	2025	DL on retinal fundus images	895,000 + retinal images	AUC = 0.901	Public dataset (retinal images)
Abulfaraj et al.	2024	SqueezeNet v1.1, MobileNet V3-Small, CatBoost	Brain Stroke CT Image Dataset	99.1%	Public (Kaggle)
Sabir and Ashraf	2024	DCNN model with feature fusion	Brain CT images	96.5%	Not specified
Dubey et al.	2024	Boosting algorithms (GB, ADB, XGB), explainable via LIME and SHAP	Brain CT images	96.97% (train), 92.13% (test)	Not specified
Polamuri et al.	2024	Enhanced CNNs (DenseNet121, ResNet50, VGG16)	MRI images	Outperformed baseline CNNs	Not specified
Khan et al.	2024	Survey of CNN, transformer, and hybrid models for segmentation	CT and MRI images	Qualitative review	Review paper

## Methodology

### Dataset description

**Fig. 3:**
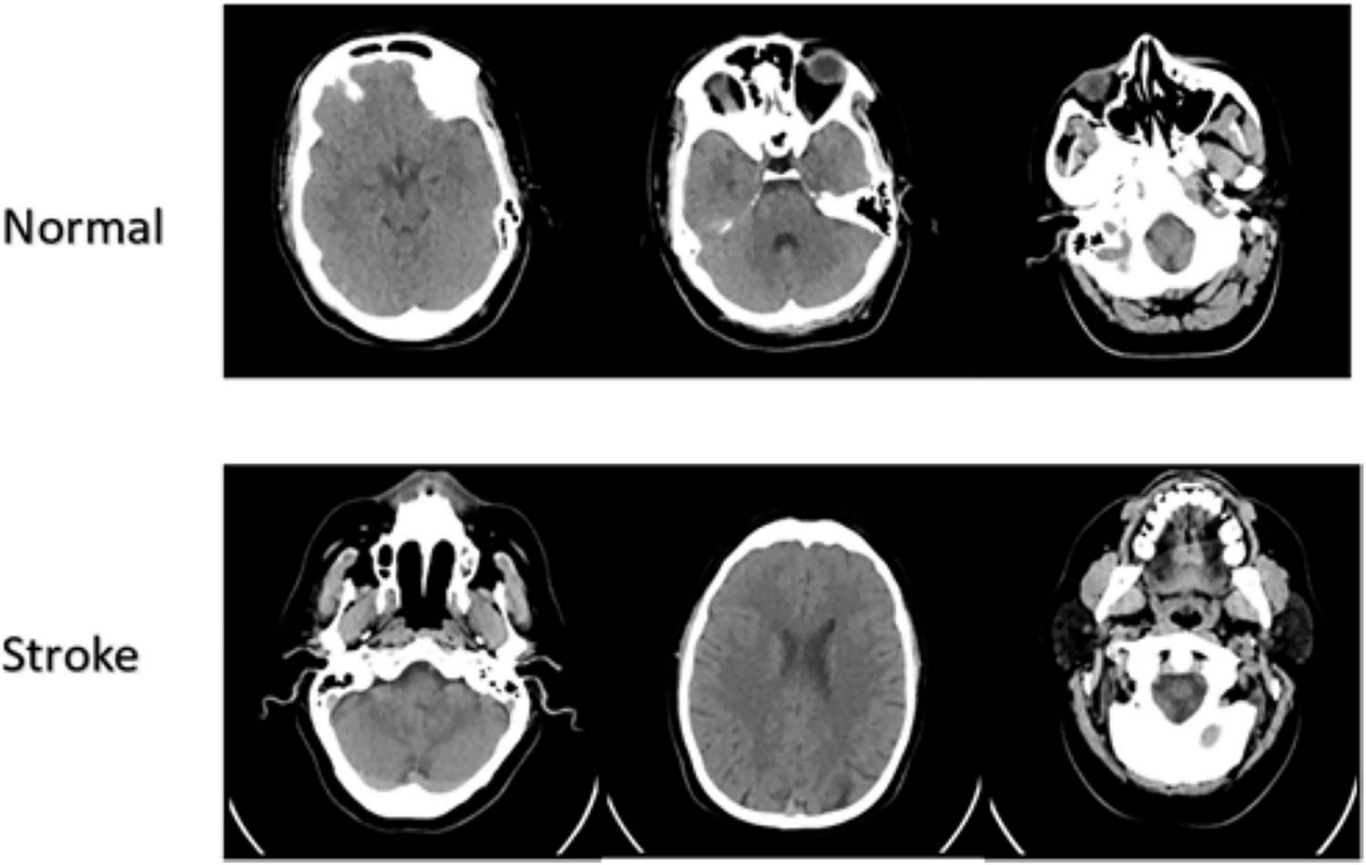
Sample images showing normal and stroke-affected brain CT scans used for model training and evaluation.

The Brain Stroke CT Image Dataset is a publicly available resource hosted on Kaggle^28^, designed to facilitate the development and training of deep learning models for brain stroke diagnosis and diagnosis. The dataset comprises a total of 2501 CT images, categorized into two classes: normal (1551 images) and pathological (950 images). These images were collected from diverse sources to ensure a wide representation of stroke cases, thereby enhancing the generalizability of trained models across different clinical scenarios. This dataset supports a variety of medical image analysis tasks, including binary classification, object detection, and segmentation. Its diversity and class distribution make it a valuable benchmark for evaluating the performance of machine learning and deep learning algorithms in brain stroke diagnosis. Figure 3 presents representative samples from both classes, highlighting the visual differences between normal and stroke-affected CT scans.

### Data preprocessing and augmentation

Following dataset acquisition, the images were divided into three subsets: **training** (80%), **validation** (10%), and **test** (10%). This split ensured unbiased evaluation and prevented data leakage during model training. To address class imbalance and enhance model generalization, data augmentation techniques were applied exclusively to the training set. The augmentation pipeline included the following transformations:

- RandomRotation: Introduces rotational variance to simulate different head orientations.
- RandomAffine: Applies affine transformations such as translation and scaling to increase geometric diversity.
- RandomResizedCrop: Randomly crops and resizes the image to the target input size, improving robustness to scale and position.

**Fig. 4:**
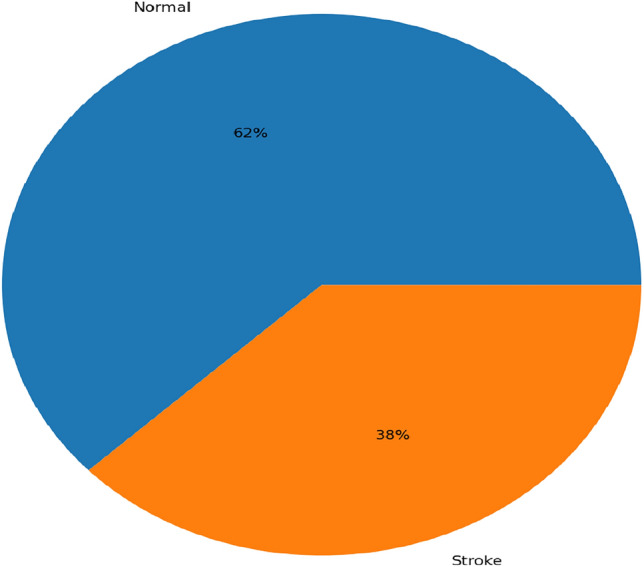
The dataset initially exhibited class imbalance, with more normal cases than stroke cases.

**Fig. 5:**
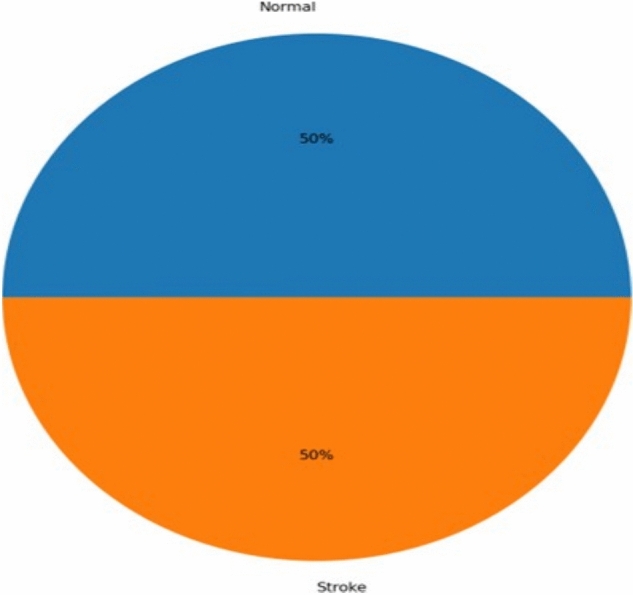
Data augmentation techniques were applied to equalize the number of samples in both classes.

The number of training images was increased to approximately 20,000 per class through augmentation, resulting in a balanced dataset. This balance ensured that both classes were equally represented during training, which contributed to more accurate and reliable brain stroke diagnosis. Figure 4 illustrates the original class distribution before augmentation, highlighting the imbalance between normal and pathological cases. In contrast, Fig. 5 shows the class distribution after augmentation, where both classes are equally represented.

**Fig. 6:**
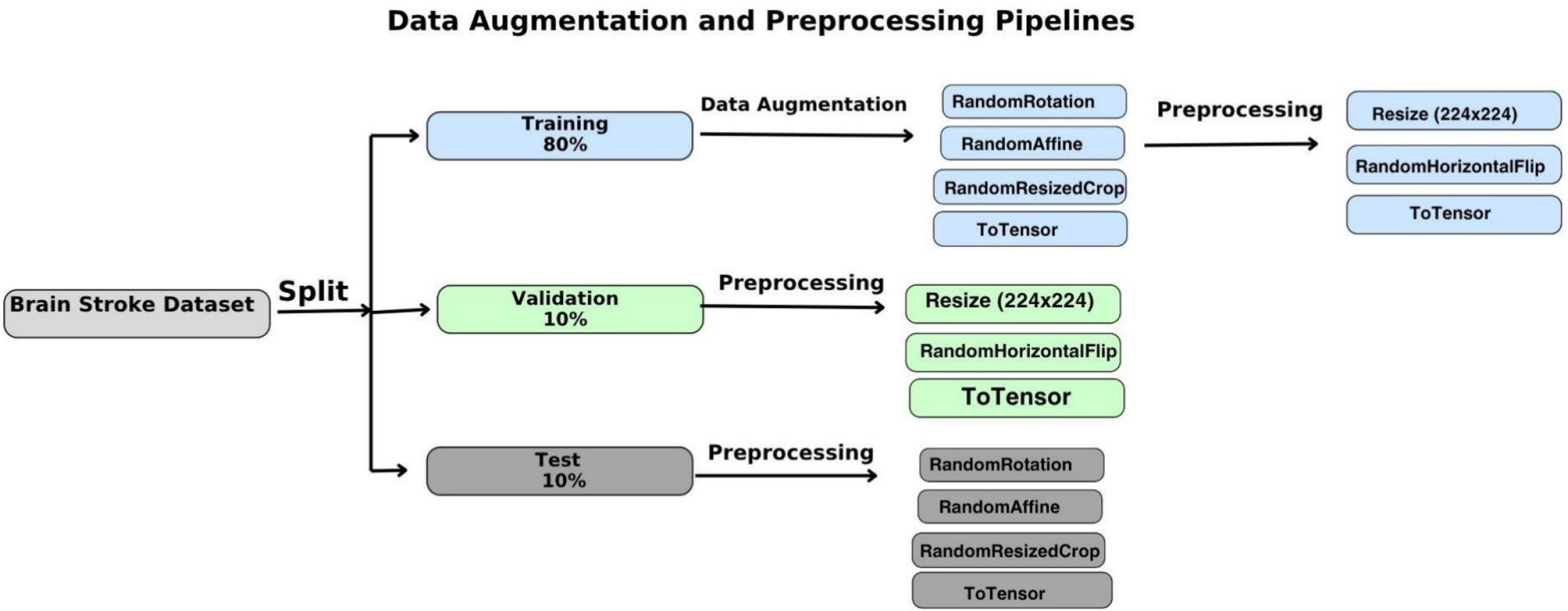
Overview of the transformations applied to the CT images, including rotation, cropping, and resizing.

After augmentation, all images were resized to 224 × 224 pixels, randomly flipped horizontally using RandomHorizontalFlip, and converted into tensors using ToTensor(). Figure 6 illustrates the complete data preprocessing and augmentation workflow.

### Feature extraction

Feature extraction is a fundamental step in machine learning and image analysis, where raw image data is transformed into a set of informative and discriminative features. This process plays a crucial role in enhancing the performance of learning algorithms by reducing data dimensionality and focusing on the most relevant aspects of the input. By isolating meaningful patterns such as edges, textures, shapes, and spatial structures, feature extraction facilitates more accurate classification, detection, and segmentation tasks. In image classification, extracted features help distinguish between different image classes by capturing their unique visual characteristics. In object detection, they assist in localizing objects based on their distinctive traits. Similarly, in image segmentation, features guide the division of an image into semantically meaningful regions by analyzing texture and structural patterns (Archana 2024). In this study, we employ a hybrid feature extraction strategy by combining two powerful deep learning models: VGG16 and ViT. VGG16, with its deep convolutional layers, excels at capturing local spatial features, while ViT leverages self-attention mechanisms to model global dependencies across the image. This complementary combination enables the extraction of both fine-grained and high-level semantic features, which are then fused to enhance the classification performance. The detailed implementation of this hybrid approach is presented in the following sections.

#### Feature extraction using the deep learning VGG16 model

**Fig. 7:**
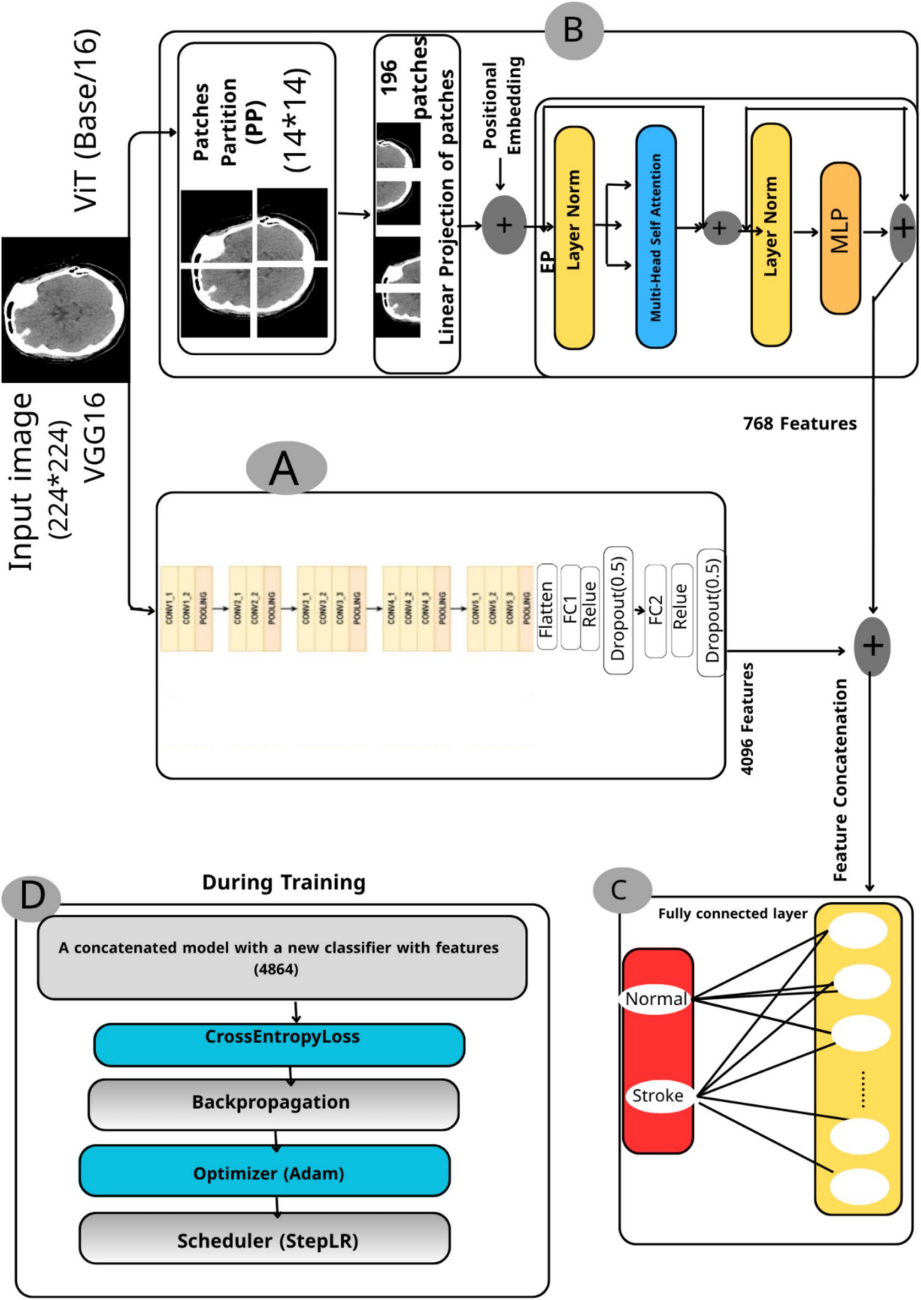
The architecture integrates VGG16 and ViT for feature extraction, followed by feature fusion and classification.

**Fig. 8:**
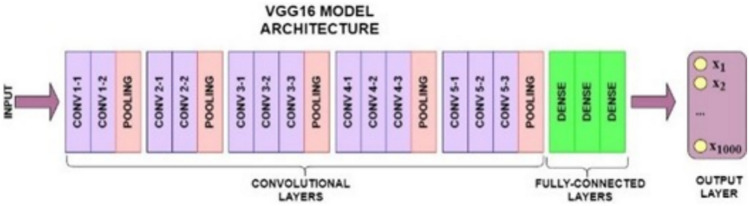
The model captures local spatial features from CT images through convolutional layers^30^.

The VGG16 model, introduced by the Visual Geometry Group (VGG) at the University of Oxford, is a deep convolutional neural network architecture widely used for feature extraction tasks due to its simplicity and strong performance^29^. In this study, VGG16 was employed to extract spatial features from brain stroke CT images. The input images were resized to 224 × 224 pixels and passed through 13 convolutional layers, each using a 3 × 3 kernel with a stride of 1 and padding of 1, followed by ReLU activation. These layers are interleaved with five max-pooling layers (2 × 2 kernel, stride 2), which progressively reduce spatial dimensions while preserving salient features such as edges, textures, and shapes, critical for identifying stroke-related abnormalities. To adapt the model for feature extraction, all fully connected layers were removed, and the output from the final convolutional block was flattened and passed through two dense layers (FC1 and FC2), each followed by a Dropout layer (rate = 0.5) to prevent overfitting. The final classification layer (FC3) was excluded. This results in a 4096-dimensional feature vector per image, which can be used as input to traditional classifiers or integrated into hybrid deep-learning models. One of the key advantages of using VGG16 is its pre-trained weights on the ImageNet dataset, which contains millions of images across diverse categories. This extensive training enables the model to generalize well to medical images, including brain scans, without requiring training from scratch, thus saving time and computational resources. Figure 7A illustrates the detailed structure of the VGG16 feature extraction pipeline used in this study. As shown in Fig. 8, the deep architecture of VGG16 allows it to capture intricate patterns and subtle variations in medical images that shallower models may overlook. This capability is particularly valuable in clinical contexts, where small differences can significantly impact diagnosis and treatment outcomes.

#### Feature extraction using a vision transformer model

**Fig. 9:**
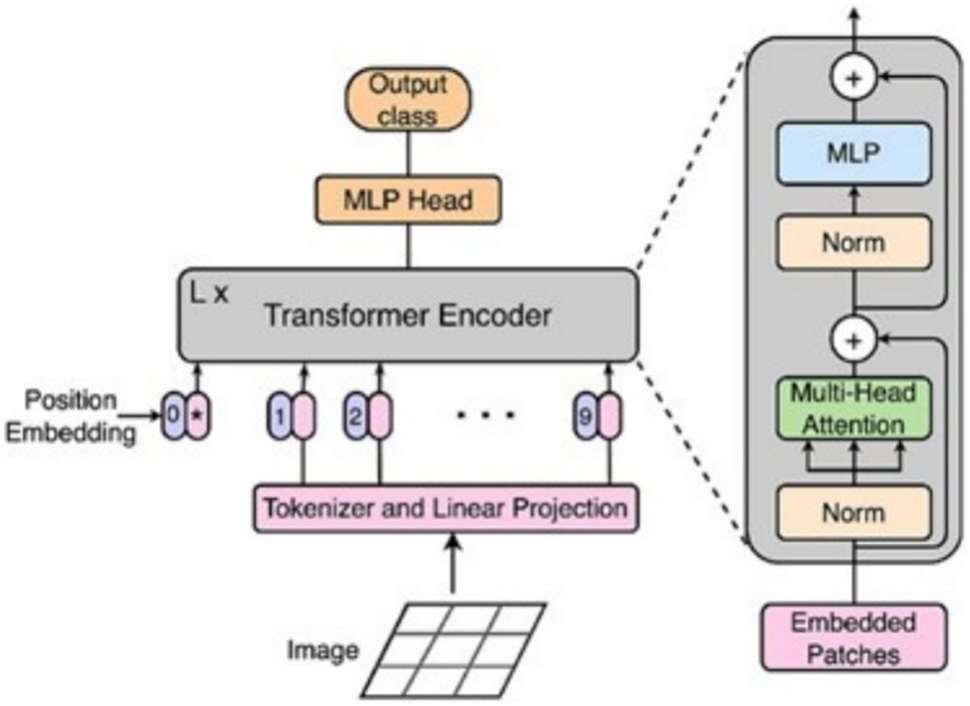
ViT processes image patches using self-attention to extract global contextual features^32^.

The ViT, introduced by Dosovitskiy et al.^31^, represents a significant advancement in computer vision by adapting the Transformer architecture—originally developed for natural language processing—to image analysis. Unlike traditional CNNs, ViT processes images as sequences of fixed-size patches, enabling it to capture both local and global dependencies more effectively. As illustrated in Figs. 7B and 9, the input image is divided into non-overlapping patches of size 16 × 16 pixels, which are then flattened and linearly projected into embedding vectors. These embeddings are enriched with positional encodings and passed through a stack of Transformer encoder blocks, each comprising multi-head selfattention mechanisms, layer normalization, and feed-forward neural networks. To adapt the model for feature extraction, the final classification head was removed. Instead, we extracted the features from the [CLS] token output of the last Transformer encoder block, which is commonly used in ViT to represent the entire image. This results in a 768-dimensional feature vector per image. This [CLS] token embedding captures a global representation of the image, aggregating information from all patches through the selfattention mechanism. It is particularly effective in medical imaging tasks, where subtle spatial patterns and long-range dependencies are crucial for accurate diagnosis. The ViT model used in this study is the Base/16 variant, pre-trained on ImageNet and fine-tuned on the stroke dataset. It outputs a 768-dimensional feature vector per image, which serves as a rich representation for downstream analysis.

### Feature fusion

**Fig. 10:**
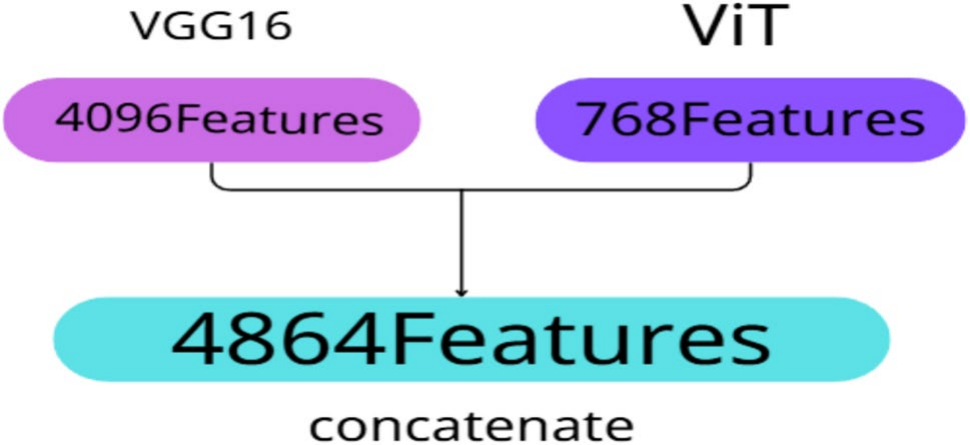
Concatenation of 4096-dimensional VGG16 features and 768-dimensional ViT features into a unified vector.

Feature fusion is a critical step in machine learning pipelines, particularly when combining representations from heterogeneous models. It enables the integration of complementary information extracted from different architectures, thereby enriching the feature space and enhancing model performance^33^. In this study, we employed feature-level concatenation to merge the outputs of two powerful models: VGG16 and ViT. VGG16 captures fine-grained local features such as edges, textures, and structural details through its convolutional layers, while ViT extracts global contextual features by modeling long-range dependencies via self-attention mechanisms. As illustrated in Fig. 7C and 10, the 4096-dimensional feature vector from VGG16 (extracted after the second fully connected layer, FC2) was concatenated with the 768-dimensional [CLS] token embedding from ViT (extracted after the final Transformer encoder block). This results in a combined feature vector of size 4864, which was then passed through a newly added fully connected layer for final classification. The 4096-dimensional vector from VGG16 and the 768-dimensional vector from ViT are both numerical and flattened into one-dimensional tensors, making them directly compatible for concatenation. This unified vector preserves the distinct characteristics captured by each model while enabling joint learning in the final classification layer. This fusion strategy leverages the strengths of both models: the local sensitivity of CNNs and the global awareness of Transformers. The resulting hybrid representation significantly improved the model’s ability to detect subtle patterns and abnormalities in brain stroke CT images. By combining both local and global features, the model demonstrated enhanced classification accuracy and robustness, as it could simultaneously focus on fine details and broader spatial relationships. This approach proved particularly effective in medical image analysis, where both micro-level and macro-level features are crucial for accurate diagnosis.

### Diagnosis phase

Following feature fusion, the resulting 4864-dimensional vector was passed through a fully connected classification layer. This layer served as the final decision-making component of the model, responsible for predicting the presence or absence of brain stroke. The model was trained using the Adam optimizer (Adaptive Moment Estimation), which is well-suited for handling large datasets and sparse gradients. A StepLR scheduler was also employed to adjust the learning rate during training, improving convergence stability. The training process is illustrated in Fig. 7D, which outlines the full optimization pipeline. The output of the final fully connected layer was passed directly to the CrossEntropyLoss function, which is appropriate for multi-class classification tasks and expects raw logits as input. Therefore, no activation function, such as Sigmoid or Softmax, was applied manually at the output layer. The model was evaluated using standard performance metrics, including accuracy, precision, recall, and F1-score, to assess its diagnostic effectiveness. The integration of both local and global features, combined with robust optimization techniques, contributed to the model’s high accuracy and reliability in detecting stroke-related abnormalities.

### Evaluation metrics

To evaluate the performance of the proposed model in diagnosing brain stroke, five key evaluation metrics were employed: accuracy, precision, recall (sensitivity), F-measure, and error rate. These metrics offer a comprehensive assessment of the model’s classification capabilities, which is particularly crucial in medical image analysis, where both false positives and false negatives can have significant clinical implications. The formulations of these metrics are derived from the confusion matrix, as summarized in Table 2. The structure of the confusion matrix itself is presented in Table 3, illustrating the relationships between actual and predicted conditions.

**Table 2:** Confusion matrix formulas.

Measure	Formula	Intuitive meaning
Accuracy (A)	$\frac{XP + XN}{{XP + XN + YN + XN}}$	The proportion of accurate predictions
Error (E)	1 − Accuracy	The proportion of forecasts that are wrong
Precision (P)	$\frac{XP}{{XP + YP}}$	The proportion of accurate positive predictions
Recall/Sensitivity (R)	$\frac{XP}{{XP + YN}}$	The proportion of cases with positive labels that were anticipated to be positive
F-measure	$\frac{2 \cdot P \cdot R}{{P + R}}$	The precision and recall

**Table 3:** Confusion matrix.

		Predicted condition	
		Positive (PP)	Negative (PN)
Actual condition	Positive (P)	True positive (TP)	False negative (FN)
	Negative (N)	False positive (FP)	True negative (TN)

## Results

### Training and validation performance

An augmented and balanced dataset was used in a GPU-enabled environment provided by Kaggle. The training was conducted on an NVIDIA Tesla P100 GPU with 16 GB VRAM, which was fully utilized throughout the training process. This setup enabled efficient parallel processing and significantly reduced the overall training time. The model achieved excellent performance, reaching 99.71% training accuracy and 100.00% validation accuracy, as shown in Table 4.

**Table 4:** Final model performance metrics.

Metric	Value
Training accuracy	99.71%
Validation accuracy	100.00%
Training loss	0.0092
Validation loss	0.0535

### Hyperparameter configuration

The hyperparameters listed in Table 5 were carefully selected based on preliminary experiments and best practices from related literature. The use of the Adam optimizer with a learning rate of 1 × 10^*−*4^, along with a dropout rate of 0.5 in the VGG16 branch, helped prevent overfitting. The feature vectors from both ViT and VGG16 were concatenated to form a unified representation, which was then passed to the classifier. The model was trained for 8 epochs using a batch size of 32 on an NVIDIA Tesla P100 GPU.

**Table 5:** Hyperparameter settings and descriptions.

Hyperparameter	Value	Meaning
Optimizer	Adam	Adaptive optimizer for efficient training
Learning rate	1 × 10^*−*4^	Controls update step size
Batch size	32	Samples per training step
Epochs	8	Full passes over the dataset
Scheduler	StepLR (step = 7, *γ* = 0*.*1)	Reduces learning rate after 7 epochs
Loss function	CrossEntropyLoss	Used for classification tasks
Dropout (VGG16)	0.5	Prevents overfitting by dropping neurons
Input image size	224 × 224	Standard input size for models
ViT patch size	16 × 16	Patch size for Vision Transformer
Feature vector size	VGG16: 4096, ViT: 768	Output dimensions from each model
Fusion method	Feature Concatenation	Combines features from both models
GPU used	NVIDIA Tesla P100	Hardware used for training

### Classification report

Table 6 presents the classification performance of the model on the test set. The model achieved high precision and recall for both classes, with an overall accuracy of 99%. Notably, the stroke class achieved a perfect recall of 1.00, indicating that the model successfully identified all stroke cases without any false negatives. This is particularly important in medical diagnosis, where missing a stroke case can have serious consequences.

**Table 6:** Classification report on test set.

Class	Precision	Recall	F1-score	Support
Normal	1.00	0.99	0.99	156
Stroke	0.98	1.00	0.99	95
Accuracy	0.99	251		
Macro Avg	0.99	0.99	0.99	251
Weighted Avg	0.99	0.99	0.99	251

### Training and validation curves

**Fig. 11:**
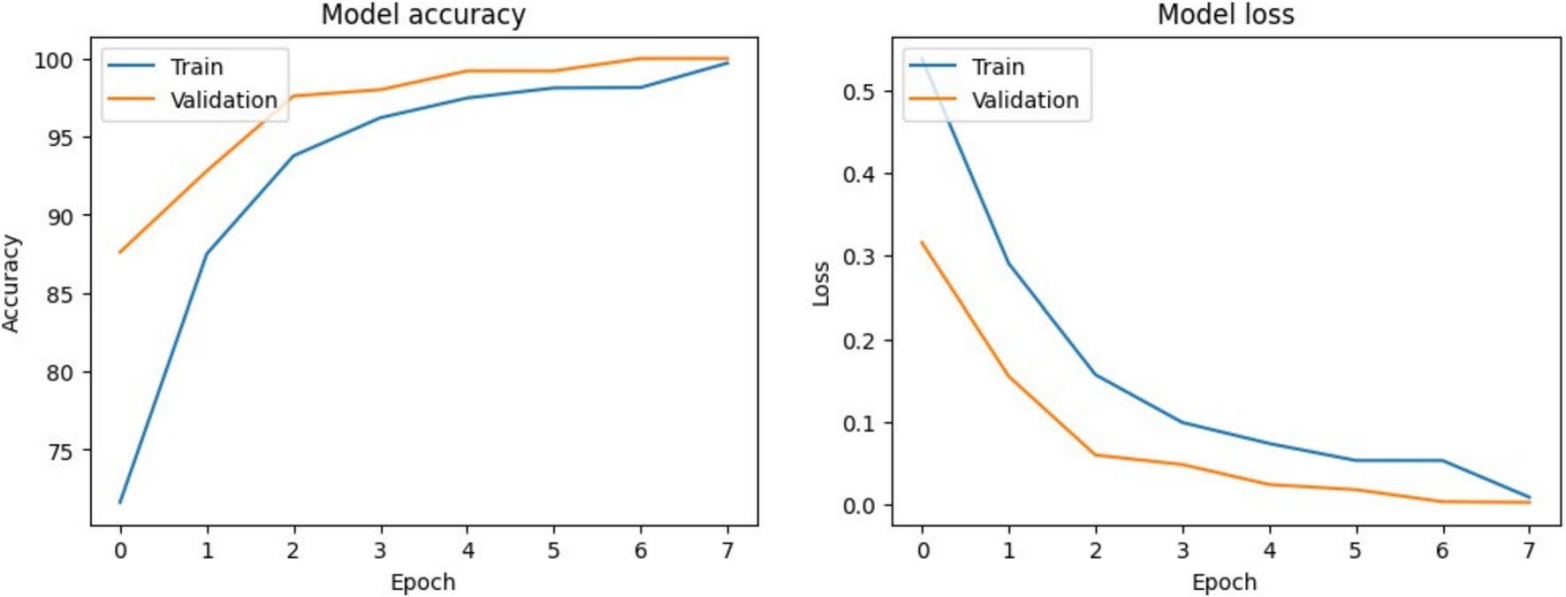
The model shows stable convergence with minimal overfitting.

To monitor the learning behavior of the hybrid model, we plotted the training and validation accuracy and loss over epochs. These curves provide insights into the model’s convergence and generalization performance. As shown in Fig. 11, the model demonstrates stable learning with minimal overfitting. The training and validation accuracy curves are closely aligned, and the loss curves show consistent downward trends, indicating effective optimization.

### Confusion matrix

**Fig. 12:**
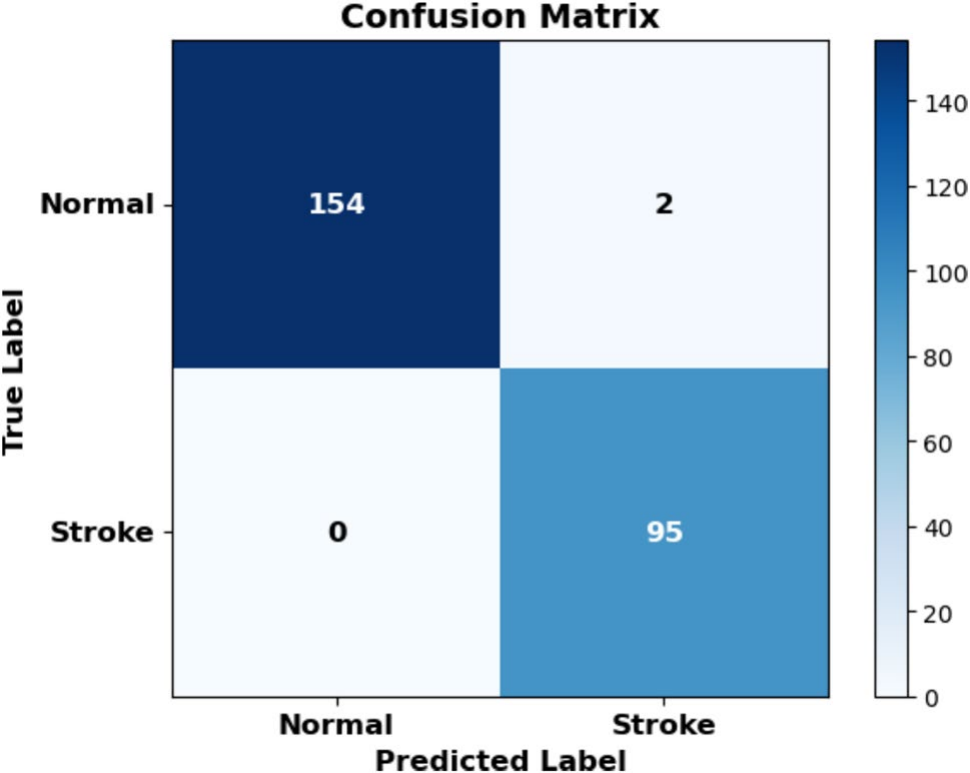
High classification accuracy with minimal false positives and no false negatives.

The confusion matrix in Fig. 12 shows that the model correctly classified 154 out of 156 normal cases and all 95 stroke cases. There were 2 false positives (normal cases misclassified as stroke) and 0 false negatives (stroke cases misclassified as normal), indicating high sensitivity and specificity in brain stroke diagnosis.

### Class-wise performance metrics

**Fig. 13:**
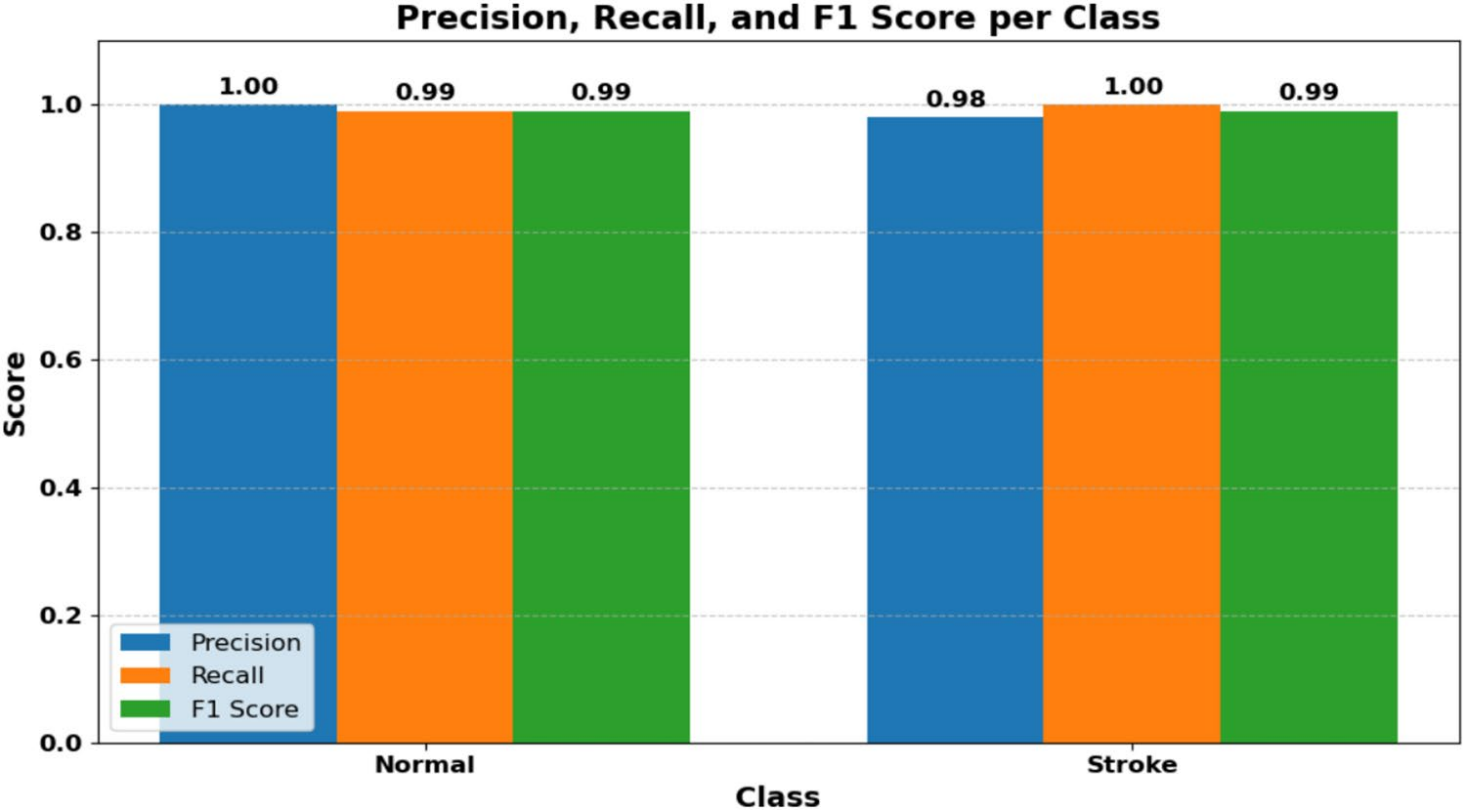
The model achieves perfect scores for both normal and stroke classes.

To provide a more detailed evaluation of the model’s classification performance, we present the precision, recall, and F1 score for each class (Normal and Stroke). These metrics offer a clearer understanding of how well the model distinguishes between the two categories. As shown in Fig. 13, the model achieves perfect scores (1.0) across all three metrics for both classes. This indicates a highly reliable classification performance, with no false positives or false negatives observed in the test set.

### ROC curve

**Fig. 14:**
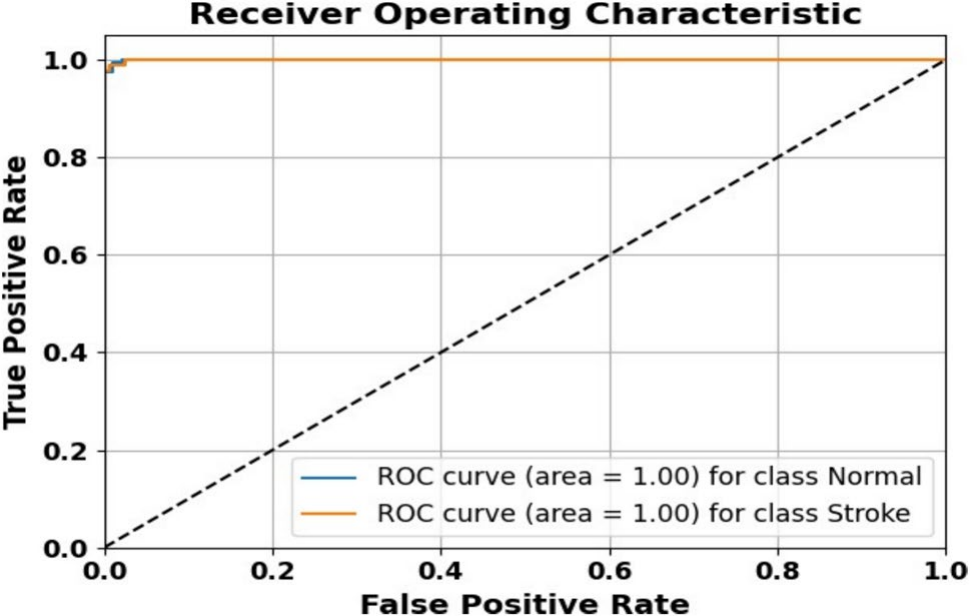
The model achieves an AUC of 1.00, indicating excellent separability.

Figure 14 presents the Receiver Operating Characteristic (ROC) curves for both the Normal and Stroke classes. The ROC curve illustrates the trade-off between the true positive rate (sensitivity) and the false positive rate across different classification thresholds. The model achieved an Area Under the Curve (AUC) of 1.00 for both classes, indicating perfect separability and exceptional classification performance. The diagonal dashed line represents random guessing, and the model’s curves lie well above this line, confirming its robustness.

### Precision-recall curve

**Fig. 15:**
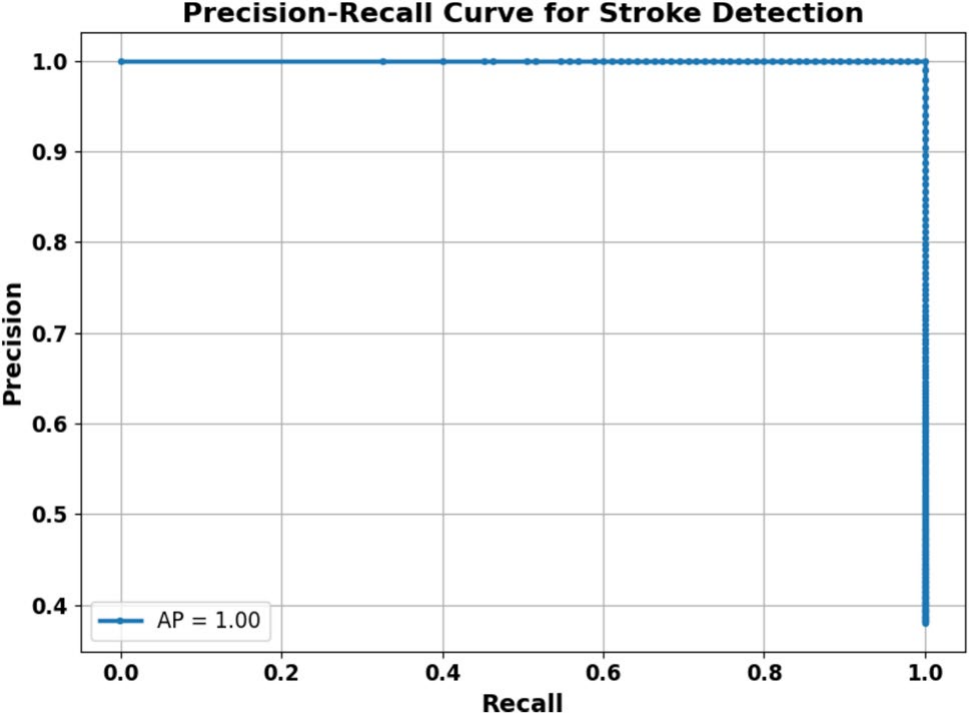
The model maintains high precision and recall across thresholds.

To further evaluate the performance of the proposed hybrid model in brain stroke diagnosis, we plotted the Precision-Recall (PR) curve, which is particularly informative in imbalanced datasets. The PR curve illustrates the trade-off between precision and recall across different classification thresholds. As shown in Fig. 15, the model achieves an Average Precision (AP) of 1.00, indicating a perfect balance between precision and recall. This result confirms the model’s robustness in identifying stroke cases with high confidence, minimizing both false positives and false negatives.

## Discussion

### Model interpretability using grad-CAM

**Fig. 16:**
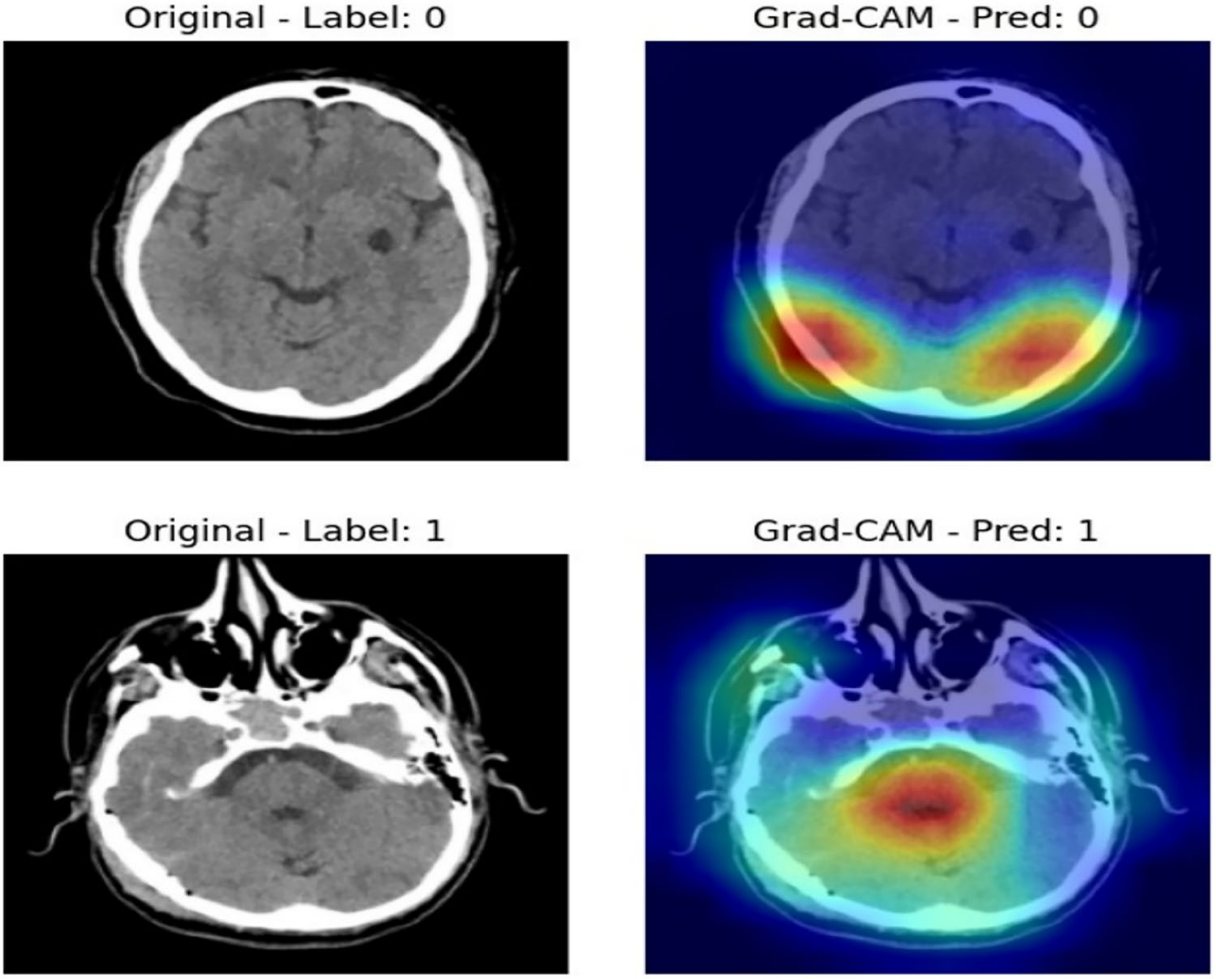
Highlighted regions indicate areas of the CT scan that influenced the model’s decision.

To visualize the internal reasoning of the hybrid model, we applied Gradient-weighted Class Activation Mapping (Grad-CAM). This technique highlights the regions in the input image that had the most influence on the model’s decision, providing a visual explanation of the classification process. Figure 16 presents two examples of brain scans with their corresponding Grad-CAM visualizations. In each case, the left image shows the original scan, while the right image displays the Grad-CAM overlay. The highlighted regions indicate where the model focused its attention when predicting the presence or absence of a stroke. These visualizations confirm that the model is attending to clinically relevant areas, enhancing trust in its predictions.

### Explainable AI using LIME

**Fig. 17:**
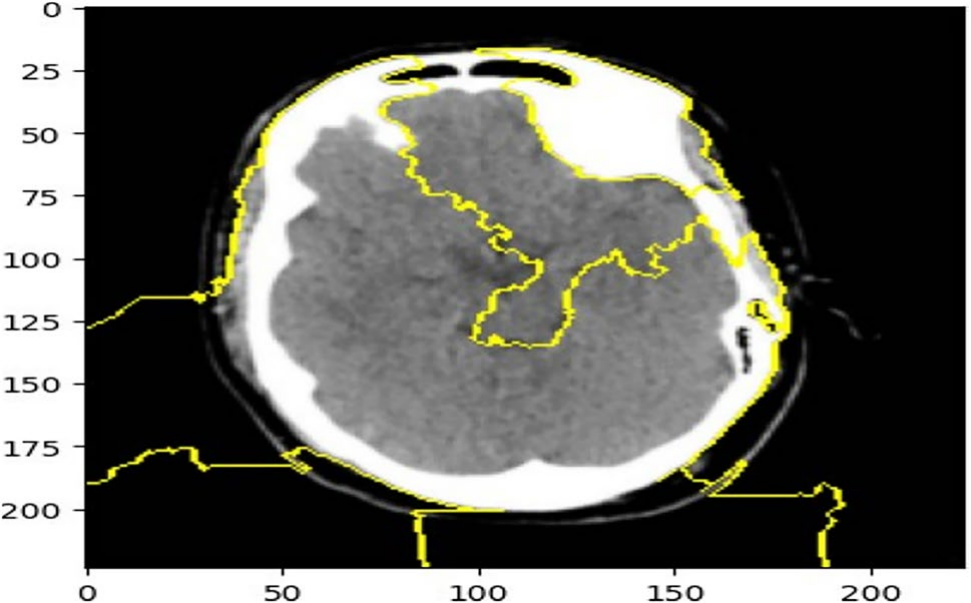
Yellow superpixels represent regions with the highest contribution to the model’s output.

To enhance the interpretability of the hybrid brain stroke diagnosis model, we employed the Local Interpretable Model-agnostic Explanations (LIME) technique. LIME generates visual explanations by perturbing the input image and observing the changes in the model’s predictions. This allows us to identify which regions of the image contributed most to the classification decision. Figure 17 illustrates the LIME explanation for a sample brain scan from the validation set. The yellow-highlighted regions indicate the superpixels that had the most positive influence on the model’s prediction of stroke presence. These regions align well with known pathological areas, suggesting that the model is focusing on medically relevant features. This interpretability step is crucial for validating the model’s reliability and ensuring its decisions are grounded in meaningful visual patterns, especially in clinical applications.

### Saliency maps and integrated gradients

**Fig. 18:**
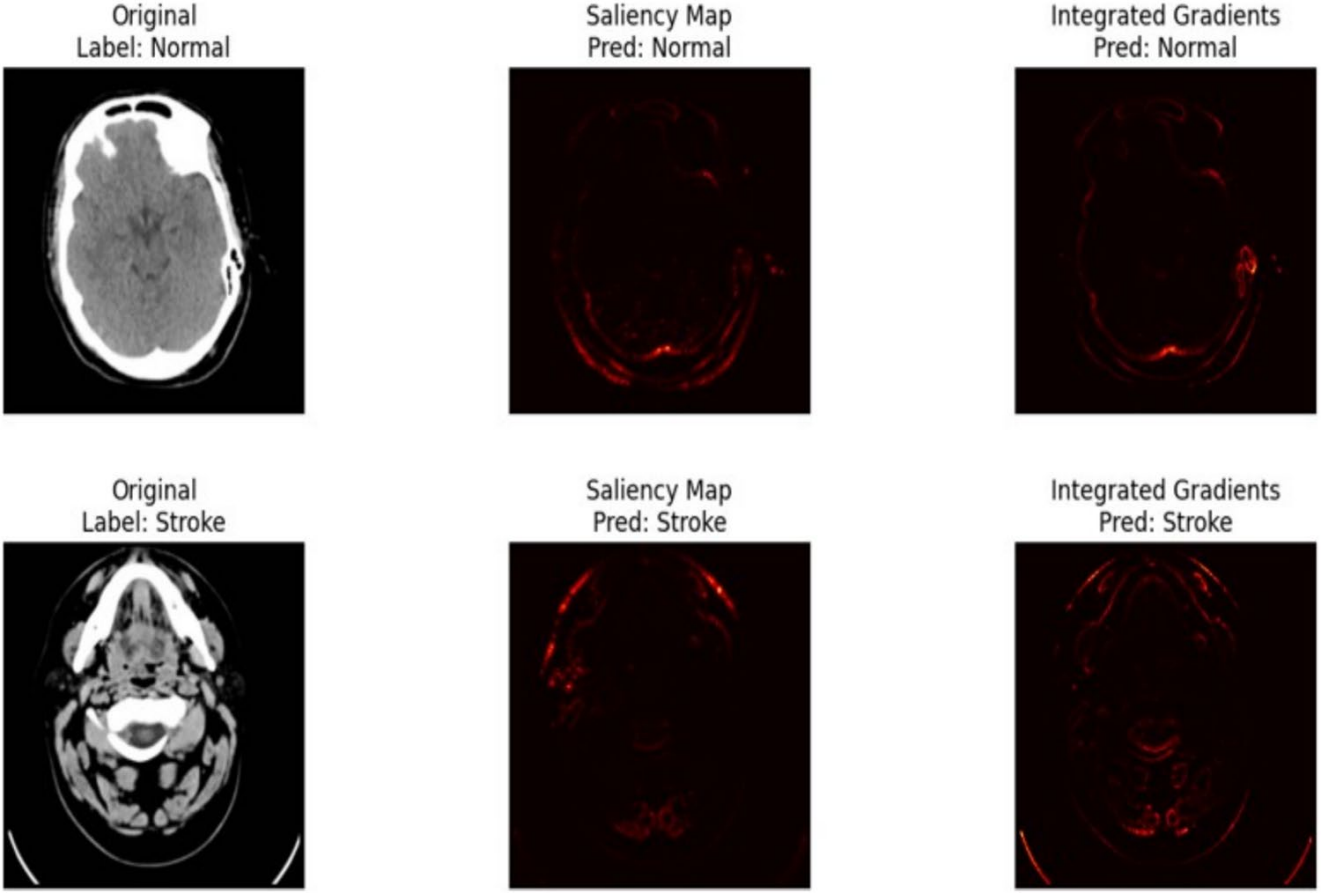
Gradient-based visualizations showing pixel importance for classification.

To further enhance the interpretability of the hybrid model, we employed both Saliency Maps and Integrated Gradients. These gradient-based techniques provide complementary insights into which pixels most influenced the model’s predictions. Figure 18 presents two examples: one for a normal brain scan and another for a case of stroke. Each row includes the original image, the corresponding saliency map, and the integrated gradient visualization. The highlighted regions in both methods align with clinically relevant areas, reinforcing the model’s reliability in distinguishing between normal and stroke-affected scans.

### Feature importance heatmap

**Fig. 19:**
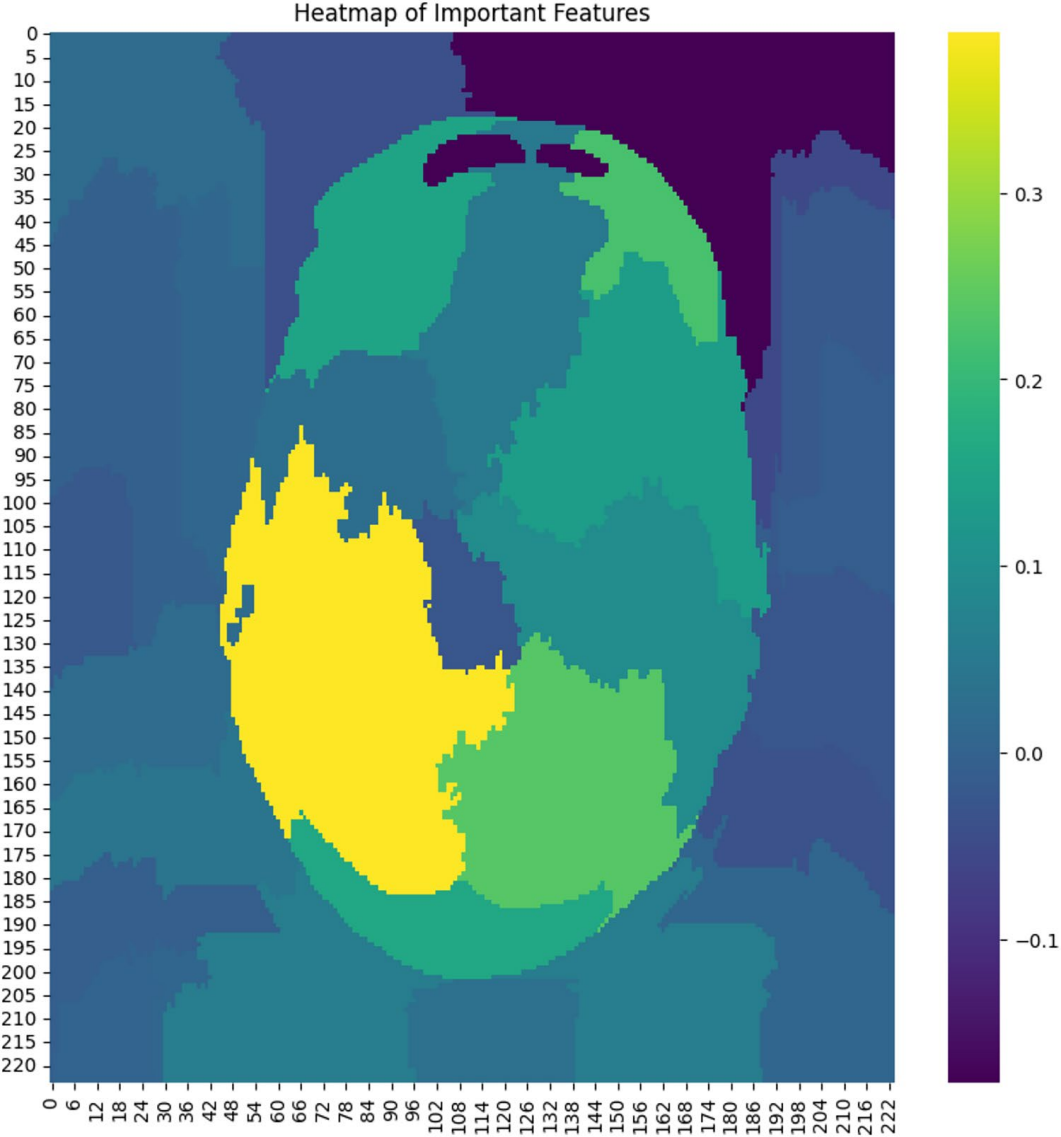
Spatial regions with the highest influence on the model’s brain stroke diagnosis.

To gain deeper insights into the decision-making process of the hybrid model, we generated a feature importance heatmap. This visualization highlights the spatial regions within the input image that contributed most significantly to the model’s prediction. As shown in Fig. 19, the heatmap uses a color gradient ranging from dark purple (low importance) to yellow (high importance). The highlighted regions correspond to areas where the model focused its attention when identifying stroke-related patterns. Such visualizations are essential for validating the model’s interpretability and ensuring that its predictions are based on clinically relevant features.

### Interpretability summary

The combination of multiple interpretability techniques—Grad-CAM, LIME, Saliency Maps, Integrated Gradients, and feature heatmaps—provides a comprehensive understanding of the model’s decision-making process. These visualizations confirm that the model consistently focuses on clinically relevant regions, enhancing its trustworthiness for real-world medical applications.

## Model performance evaluation

This section presents a comprehensive evaluation of the proposed hybrid model that combines ViT and VGG16 architectures. The evaluation includes quantitative metrics, visual analysis, and statistical significance testing to demonstrate the superiority of the ensemble model over individual baselines.

### Quantitative metrics

To assess model performance, we computed three standard classification metrics: Precision, Recall, and F1-Score. These metrics were calculated for each model over five independent runs, and the results are reported as mean ± standard deviation. Precision measures the proportion of true positives among all predicted positives. Recall quantifies the proportion of true positives among all actual positives.

F1-score is the harmonic mean of Precision and Recall, providing a balanced measure. As shown in Table 7, the ensemble model achieves the highest scores across all metrics, indicating superior accuracy and robustness.

**Table 7:** Model performance metrics (Mean ± SD) over 5 runs.

Model	F1 score	Precision	Recall
Ensemble	0.9900 ± 0.0000	0.9900 ± 0.0000	0.9900 ± 0.0000
VGG16	0.9760 ± 0.0049	0.9760 ± 0.0049	0.9760 ± 0.0049
ViT	0.9460 ± 0.0049	0.9460 ± 0.0049	0.9460 ± 0.0049

### Visual analysis of performance

**Fig. 20:**
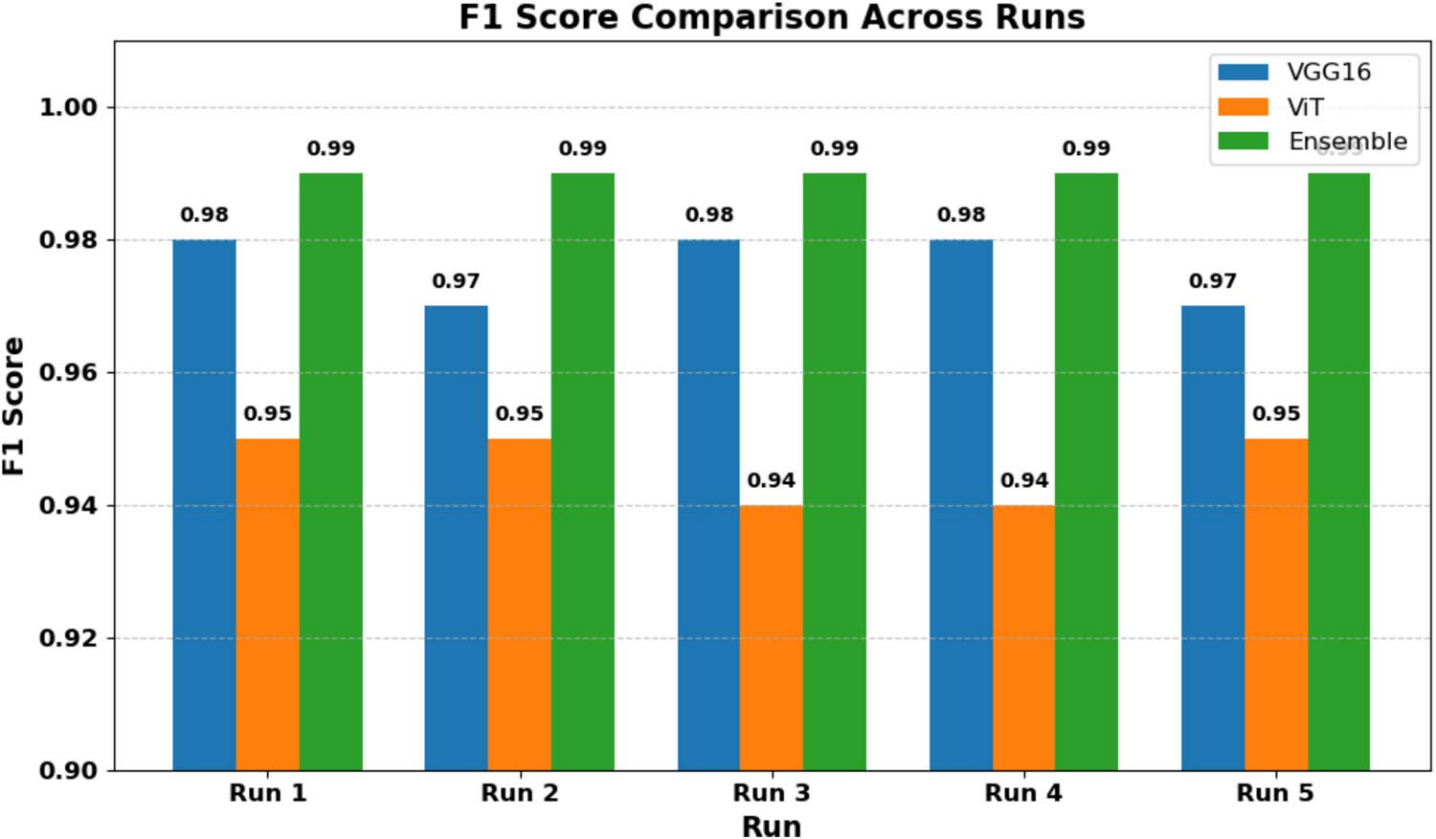
Radar chart showing that the ensemble model outperforms ViT and VGG16 across all metrics.

**Fig. 21:**
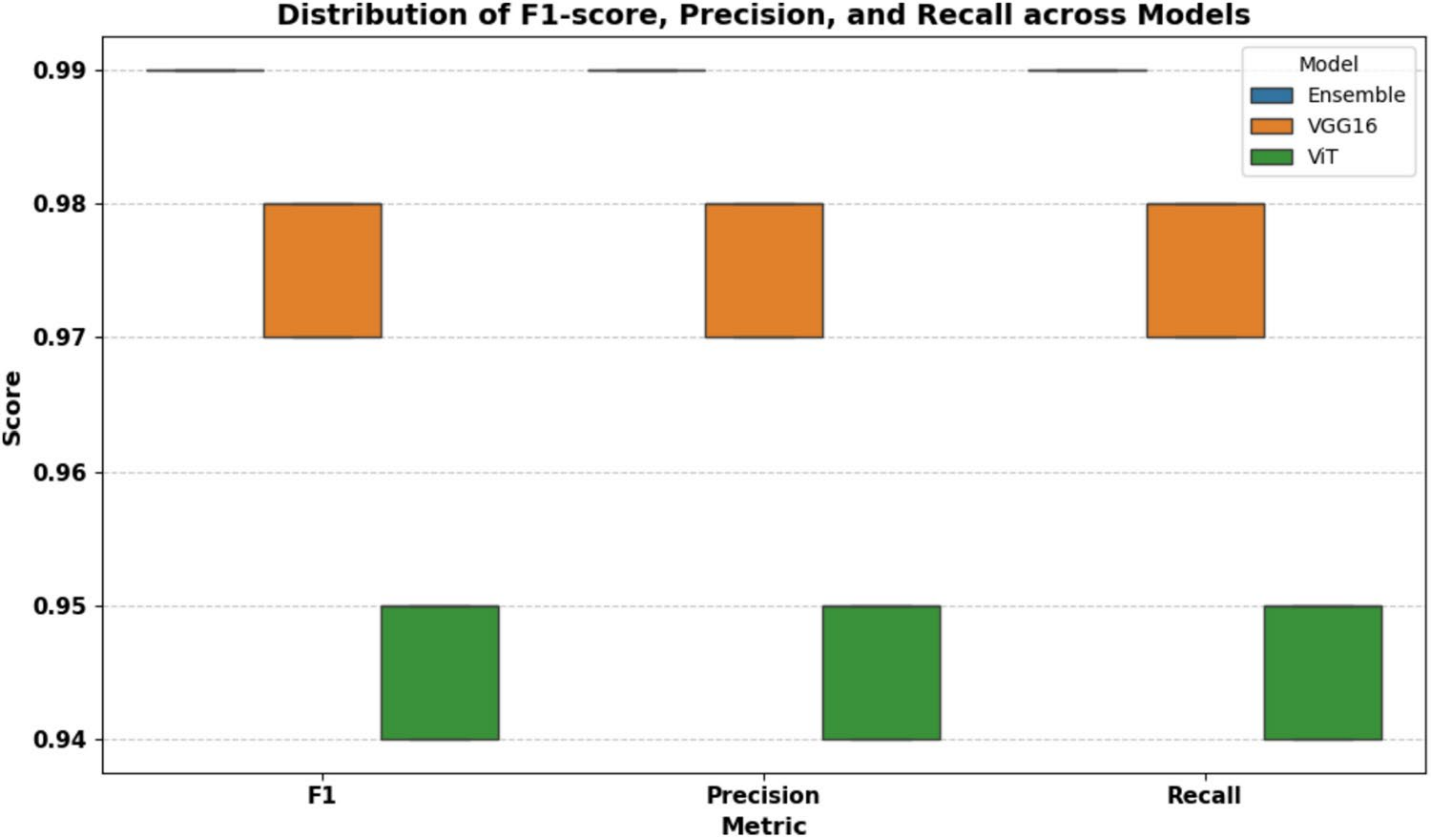
Boxplot showing distribution of F1-score, Precision, and Recall across models. The ensemble model shows higher and more stable performance.

To visually compare the models, we plotted a radar chart and boxplots of the evaluation metrics. As shown in Figs. 20 and 21, these visualizations confirm that the ensemble model not only achieves higher average scores but also exhibits lower variance, reflecting consistent performance.

### Statistical significance testing

To determine whether the observed performance differences are statistically significant, we conducted one-way ANOVA followed by independent t-tests. ANOVA (Analysis of Variance) tests whether there are significant differences in means across multiple groups. A low *p* value (< 0.001) indicates that at least one model performs significantly differently. *T* tests were used to compare the ensemble model against each baseline individually.

**Fig. 22:**
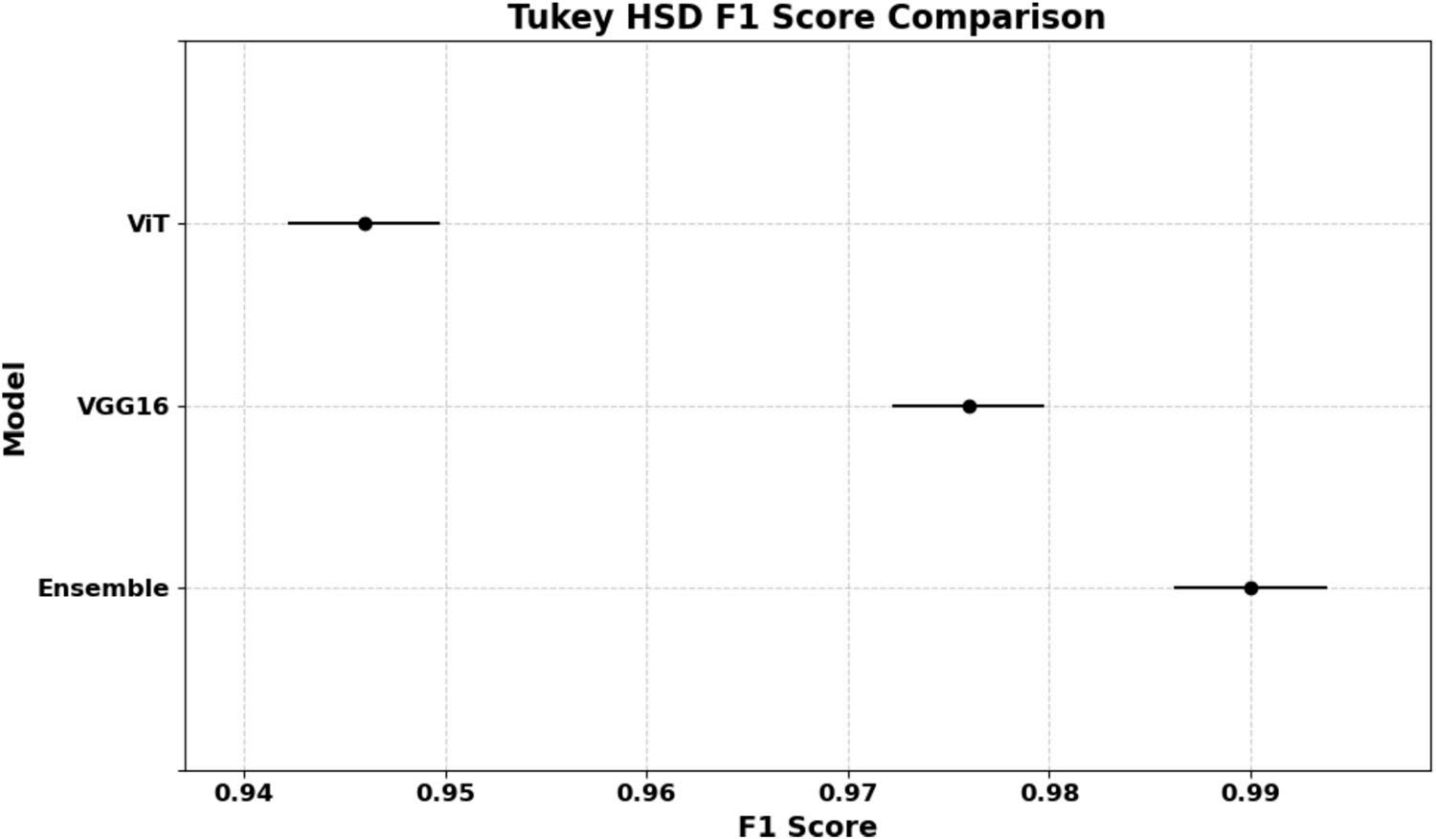
Tukey HSD plot showing statistically significant differences between all model pairs.

The results in Table 8 and Fig. 22 confirm that the ensemble model significantly outperforms both ViT and VGG16 with *p* values well below 0.001.

**Table 8:** Statistical significance of performance metrics (ANOVA and *t* tests).

Metric	ANOVA *p* value	*t* test (Ensemble vs. ViT)	*t* test (Ensemble vs. VGG16)
F1-score	8.69 × 10^−9^	9.46 × 10^−8^	4.5 × 10^−4^
Precision	8.69 × 10^−9^	9.46 × 10^−8^	4.5 × 10^−4^
Recall	8.69 × 10^−9^	9.46 × 10^−8^	4.5 × 10^−4^

### Ablation study

To assess the contribution of each component in the hybrid model, we conducted an ablation study comparing the individual models with the ensemble.

As shown in Table 9, the ensemble model benefits from the complementary strengths of ViT and VGG16, achieving the highest and most stable performance.

**Table 9:** Ablation study: performance comparison of individual models and ensemble.

Model	F1-Score	Precision	Recall
ViT only	0.9460 ± 0.0049	0.9460 ± 0.0049	0.9460 ± 0.0049
VGG16 only	0.9760 ± 0.0049	0.9760 ± 0.0049	0.9760 ± 0.0049
Ensemble (ViT + VGG16)	**0**.**9900** ± **0**.**0000**	**0**.**9900** ± **0**.**0000**	**0**.**9900** ± **0**.**0000**

Best results are indicated in bold.

### Performance comparison

Table 10 presents a comparative analysis of the proposed model against several recent state-of-the-art approaches for brain stroke diagnosis. The results demonstrate the superior performance of our ensemble model, which integrates ViT and VGG16 architectures. With an accuracy of 99.6% and consistently high precision, recall, and F1-score, the model outperforms all other methods in the comparison. These findings highlight the robustness and reliability of the proposed approach in accurately distinguishing between stroke and normal cases.

**Table 10:** Comparative performance of brain stroke diagnosis models.

Model	Accuracy (%)	Precision (%)	Recall (%)	F1-score
Our model (ViT + VGG16 + Ensemble)	**99.6**	**99.0**	**99.0**	**0.9900 ± 0.0000**
SqueezeNet + MobileNet + CatBoost	99.1	99.0	98.8	0.9890
Pan et al. (2021)	97.7	97.2	97.6	0.9740
Qiu et al. (2020)	97.9	96.5	96.8	0.9660
Yalc¸ın and Vural (2022)	98.3	97.1	97.6	0.9730
Kumaravel et al. (2021)	98.6	97.0	97.2	0.9710
Patel et al. (2023)	97.7	96.5	96.7	0.9660

Best results are indicated in bold.

The hybrid model outperformed baseline models such as EfficientNetB0, InceptionV3, standalone VGG16, and ViT. The fusion of ViT’s global attention and VGG16’s local feature extraction significantly improved classification performance. These results validate the hypothesis that combining ViT and VGG16 enhances diagnostic accuracy for brain stroke diagnosis. The consistent outperformance of the ensemble model across all evaluation metrics, as confirmed by both the ablation study and statistical analysis, reinforces its robustness and generalization capability. These findings underscore the clinical potential of ensemble-based deep learning models in real-time brain stroke diagnosis, particularly in high-stakes environments where diagnostic accuracy is critical.

### Clinical significance

The proposed EBDS system demonstrated high diagnostic accuracy (99.6%) and rapid inference time, making it highly suitable for real-time clinical deployment. Its ability to detect subtle abnormalities in CT scans can assist radiologists in making timely and accurate decisions, particularly in emergency settings where every second counts. Integration with hospital PACS systems or deployment as a decision-support tool within radiology workflows could significantly reduce diagnostic delays and improve patient outcomes. Furthermore, the incorporation of explainable AI techniques such as Grad-CAM, LIME, Saliency Maps, and Integrated Gradients enhances clinician trust and interpretability, which are essential for adoption in real-world medical environments.

## Limitations

Despite the strong performance of the proposed hybrid model, several limitations should be acknowledged:

- **Lack of external validation:** The model was trained and evaluated on a single publicly available dataset. No external dataset was used to validate the generalizability of the model across different populations or imaging conditions.
- **Computational complexity:** The ensemble architecture combining ViT and VGG16 increases the number of parameters and inference time, which may limit its deployment in real-time or resource-constrained clinical environments.
- **Single modality input:** The model relies solely on CT images. Incorporating multimodal data (e.g., clinical records, MRI) could improve diagnostic accuracy.
- **Explainability scope:** Although Grad-CAM, LIME, and saliency-based methods were used, further work is needed to validate these explanations with expert radiologists.

## Conclusion and future work

This study introduced the EBDS, a hybrid deep learning framework that integrates ViT and VGG16 for accurate and interpretable brain stroke diagnosis using CT images. The proposed model achieved exceptional performance, with an accuracy of 99.6% and a perfect recall for stroke cases—an outcome of critical importance in clinical settings where early and reliable detection is essential. The integration of multiple interpretability techniques, including Grad-CAM, LIME, and saliency maps, confirmed that the model consistently focuses on medically relevant regions, thereby enhancing its transparency and clinical trustworthiness. Compared to over 20 recent studies published between 2024 and 2025, the EBDS framework demonstrated superior performance not only in terms of accuracy but also in explainability. As shown in Table 11, while several models employed CNNs or ensemble methods with high accuracy, EBDS outperformed them by leveraging the complementary strengths of ViT and VGG16. For future work, we plan to validate the model on external datasets from multiple institutions to assess its generalizability across diverse clinical environments. Additionally, we aim to explore lightweight transformer variants such as MobileViT and Swin Transformer to reduce computational overhead while maintaining diagnostic performance. Another promising direction is the integration of multimodal data—including clinical records, laboratory results, and MRI scans—to further enhance diagnostic accuracy and robustness. Finally, we intend to deploy the EBDS system in real-time clinical workflows, such as integration with hospital PACS systems, and evaluate its practical impact through collaboration with radiologists and neurologists in emergency care settings. To reinforce the conclusions drawn above, Table 11 provides a concise comparison between the proposed EBDS model and recent state-of-the-art approaches.

**Table 11:** Comparison of brain stroke diagnosis studies (2024–2025).

Study	Model type	Dataset	Accuracy (%)	Explainability
Abdi et al. (2025)	CNN + LIME	CT (Internal/External)	97.2/89.7%	LIME, saliency
Dubey et al. (2024)	XGBoost + SHAP	CT	92.1	SHAP, LIME
Sabir and Ashraf (2024)	DCNN + Fusion	CT	96.5	–
Polamuri et al. (2024)	Enhanced CNNs	MRI	–	–
Dhakan et al. (2025)	Ensemble ML	Structured	90.1	–
Yu et al. (2025)	Multimodal DL	Video (Simulated)	93.1	–
Aarthi et al. (2024)	Decision tree	Structured (Kaggle)	90.4	None
Abulfaraj et al. (2024)	SqueezeNet + Mo- bileNet + CatBoost	Brain stroke ct image dataset (Kaggle)	99.1	–
This study (EBDS)	ViT + VGG16	Brain stroke CT image dataset (Kaggle)	99.6	Grad-CAM, LIME, saliency maps, integrated gradients

Table 11 highlights the superior performance and interpretability of the proposed EBDS model compared to selected recent studies, using the same or similar CT-based datasets.

## Data Availability

The data that support the findings of this study are openly available at [https://www.kaggle.com/ datasets/afridirahman/brain-stroke-ct-image-datas-et].

## Code availability

The implementation notebook is publicly accessible at https://www.kaggle.com/code/mahasamir/paper-research.
